# Typhoid Fever

**Published:** 1901-08-24

**Authors:** 


### TYPHOID FEVER.

In opening a discussion at Cheltenham on enteric fever Dr. Houston commenced by saying that he was greatly struck with the fact that notwithstanding the progress of science and the great efficiency of the public health service, we find ourselves at the beginning of a new century unable to stamp out a disease which is, nevertheless, still classed as a preventable one; and after careful review of the present position of our knowledge in regard to the natural history, the modes of dissemination, and the prophylaxis of the disease, he ended by the remark that the discoveries of recent times have but confirmed the wisdom of the teaching of epidemiologists at a time when the science of bacteriology was unknown or only in its infancy. In illustration of this he quoted from the late Sir Richard Thorne Thorne's introduction to the Local Government Board report on Oyster Culture in Relation to Disease as follows:—"Long before Koch's cholera vibrio or Eberth's typhoid bacillus had been even heard of, the remarkable progress made in the prevention of cholera and of enteric fever in this country was the result of the recognition of the fact that water and food, amongst other things, which had been subject to contamination by human excreta, served as channels for the communication of the specific contagia of those diseases to man." The general measures which have governed sanitarians in the past remain the same to-day. These are (1) to protect our water supplies from excremental pollution; (2) to secure that all articles of food are free from objectionable contamination; (3) to secure that our drains and sewers are kept in good order; and (4) to disinfect the stools and urine and soiled linen of enteric fever patients. In regard to the latter point he urged the administration of urotropine throughout the whole course of the disease and for some weeks afterwards in all cases of enteric fever. When it is remembered that every fourth case may present this serious complication, that every drop of such urine may contain not only thousands but millions of typhoid bacilli, and that soiling of the linen with urine is impossible to avoid, the extreme importance of the subject will be readily admitted. In nearly all cases, he says, urotropine may be given in 30-grain doses for months without any ill effects, and without prejudice to other medicines and the usual treatment of the disease. It is true, he adds, that in a few cases hæmaturia and albuminuria have been caused by its use, but on ceasing the administration of the drug these symptoms have rapidly disappeared. On public health grounds then he urged in the strongest manner "that this valuable remedy should be administered in all cases of enteric fever." As to the utility of anti-typhoid inoculations he was by no means so positive. In India, the results have been of an encouraging nature, but as regards South Africa, while some competent authorities have spoken very favourably of the results, others equally competent have arrived at an opposite conclusion. However the results are interpreted it cannot, he says, be expected that preventive inoculation has any great future before it in this country. It is chiefly in the case of people going abroad and as regards our troops serving in India, South Africa, and other places where typhoid fever is very prevalent, that such inoculations would be most serviceable, if it should be shown that they can do what is claimed for them.

